# Recent Advances and Perspectives of Molecularly Imprinted Polymer-Based Fluorescent Sensors in Food and Environment Analysis

**DOI:** 10.3390/nano9071030

**Published:** 2019-07-18

**Authors:** Guangyang Liu, Xiaodong Huang, Lingyun Li, Xiaomin Xu, Yanguo Zhang, Jun Lv, Donghui Xu

**Affiliations:** 1 Institute of Vegetables and Flowers, Chinese Academy of Agricultural Sciences, Key Laboratory of Vegetables Quality and Safety Control, Ministry of Agriculture and Rural Affairs of China, Beijing 100081, China; 2 Laboratory of Quality & Safety Risk Assessment for vegetable Products, Ministry of Agriculture and Rural Affairs of China, Beijing 100081, China

**Keywords:** molecularly imprinted polymer, fluorescence sensor, food quality and safety, rapid detection, molecular recognition

## Abstract

Molecular imprinting technology (MIT), also known as molecular template technology, is a new technology involving material chemistry, polymer chemistry, biochemistry, and other multi-disciplinary approaches. This technology is used to realize the unique recognition ability of three-dimensional crosslinked polymers, called the molecularly imprinted polymers (MIPs). MIPs demonstrate a wide range of applicability, good plasticity, stability, and high selectivity, and their internal recognition sites can be selectively combined with template molecules to achieve selective recognition. A molecularly imprinted fluorescence sensor (MIFs) incorporates fluorescent materials (fluorescein or fluorescent nanoparticles) into a molecularly imprinted polymer synthesis system and transforms the binding sites between target molecules and molecularly imprinted materials into readable fluorescence signals. This sensor demonstrates the advantages of high sensitivity and selectivity of fluorescence detection. Molecularly imprinted materials demonstrate considerable research significance and broad application prospects. They are a research hotspot in the field of food and environment safety sensing analysis. In this study, the progress in the construction and application of MIFs was reviewed with emphasis on the preparation principle, detection methods, and molecular recognition mechanism. The applications of MIFs in food and environment safety detection in recent years were summarized, and the research trends and development prospects of MIFs were discussed.

## 1. Introduction

Molecular imprinting technology (MIT) is a preparation technology based on the interaction and principle of antibody and antigen as well as enzyme and substrate, which is developed to synthesize three-dimensional crosslinked polymers with specific molecular recognition ability [1,2,3]. Molecularly imprinted polymers (MIPs) demonstrate many advantages, such as good selective adsorption, strong affinity, simple preparation, strong stress resistance, and low cost. They demonstrate significant application prospects in the fields of solid phase extraction, chemical biomimetic sensing technology, chromatographic separation, and mimic enzymes [4,5,6]. Traditional MIPs possess good specific recognition performance; however, they lack signal output ability during analysis and detection. Therefore, they need to be used in combination with instrumental confirmation methods [7]. The fluorescence sensor generally comprises a recognition unit and a signal output unit. Molecularly imprinted fluorescence sensors (MIFs) can be constructed by introducing the fluorescent material into the molecularly imprinted polymer synthesis system, which can realize specific recognition and fluorescence detection of the target [8,9]. MIFs have emerged into a research hotspot in the fields of medicine, environment, and food safety sensing analysis [10,11,12,13].

In the past decade, several food and environment safety incidents had occurred, which resulted in food safety becoming the focus of global attention [14,15]. Food safety is closely related to human health, economic development, and social stability and is a major issue with respect to national economy and people’s livelihood [16]. Currently, the food safety detection technology includes instrument confirmation technology and rapid detection technology. The instrument validation techniques include gas chromatography [17], liquid chromatography [18], capillary electrophoresis [19], supercritical fluid chromatography [20], gas chromatography-mass spectrometry [21,22], and liquid chromatography-mass spectrometry [23,24,25]. However, the instrumentation method demonstrates drawbacks of a complex sample pretreatment process, high costs, complex operation, long detection time, and large-scale expensive equipment. This method is not capable of achieving rapid measurement on-site detection, thereby requiring professional operators. In contrast, the rapid detection technology is simple, rapid, low-cost, selective, and demonstrates high specificity [26,27,28]. Therefore, it is suitable for rapid screening and monitoring of food quality and safety [29,30,31]. In recent years, the rapid detection technology of food quality and safety hazard factors has developed rapidly. These developments mainly include the ultraviolet visualization technology [32], fluorescence sensing technology [33], Raman technology [34], biological immune technology [35], and electrochemical technology [36].

MIFs not only demonstrate the advantages of specific recognition and specific adsorption of molecular imprinting but also possesses the high sensitivity and high selectivity of fluorescent materials. This characteristic is important in integrating the recognition unit and signal output unit efficiently in the rapid detection of food quality and safety, and demonstrates broad application prospects [37,38]. In this review, the preparation of MIFs, the detection methods and molecular recognition mechanisms are summarized. The application status of MIFs in the rapid detection of food quality and safety hazard factors (agricultural and veterinary drug residues, heavy metals, and environmental organic pollutants) is analyzed. The research focus and development trend of the MIFs is discussed.

## 2. Preparation of MIPs

**Figure 1:**
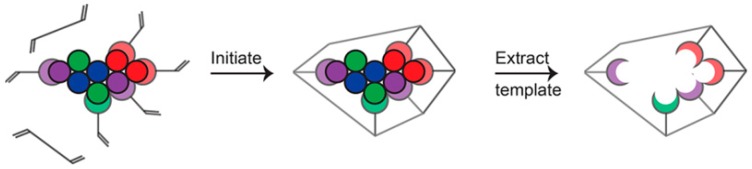
Illustration schematic of the preparation principle of molecularly imprinted polymers (MIPs). Reprinted with permission from [40], copyright (2017) American Chemical Society.

MIPs are a class of polymeric materials formed using template molecules and functional monomers through covalent or non-covalent bonds to create preassemblies. Under the action of cross-linking agents and initiators, they are then completely matched with the template molecules in shape and structure [39]. The preparation principle of MIPs is shown in Figure 1 and the process is generally divided into three steps [40]: (1) template molecules and functional monomers are preassembled in suitable solvents, and the host and guest recognize each other to form stable supramolecular complexes [41,42] with multiple specific recognition sites and specific spatial arrangement. (2) A crosslinking agent and an initiator are added into the preassembly solution, the process of photopolymerization or thermal polymerization is initiated by the initiator, and a highly crosslinking polymerization is performed around the template-functional monomer supramolecular complex to form a rigid polymer with a three-dimensional spatial structure [43]. (3) Elute the template molecules embedded in the polymer with an eluent (mostly alkyd or alkali alcoholic solution) to obtain a MIP [44] with three-dimensional holes that can be perfectly matched to the template molecule. The imprinted cavities can selectively bind to the template molecules again and demonstrate a specific recognition effect on the template molecules.

## 3. Construction of a MIFs

**Figure 2:**
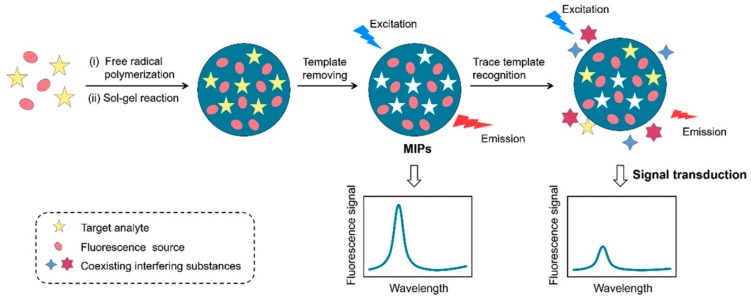
The preparation process of molecularly imprinted fluorescence sensors (MIFs) based on embedding fluorescent materials into MIPs. Reprinted with permission from [45], copyright (2018) Elsevier.

The fluorescence sensor can convert the recognition between the molecular recognition unit and target into a fluorescence response signal and detect the concentration of the target by monitoring the fluorescence intensity. An MIFs was constructed by introducing fluorescent materials into the MIPs synthesis system. Figure 2 presents the preparation process of MIFs based on embedding fluorescent materials into MIPs [45]. MIFs are highly selective, highly sensitive, specific, and stable compared to conventional sensors [46], and have been widely used to detect many kinds of pollutants [47,48,49,50,51].

### 3.1. Detection Mechanism

There are two possible directions to construct MIFs, including direct fluorescence detection and indirect fluorescence detection, according to the varied properties of the sample [52]. Direct fluorescence detection is employed to detect the fluorescence intensity of MIPs directly, when the target (template molecule) can generate a fluorescence signal after recognition and adsorption. Indirect fluorescence detection includes two steps: (1) The fluorescent molecularly imprinted polymer (FIP) (fluorescent functional monomer imprinted cavity or embedded fluorescent material) is prepared using fluorescent materials, and the fluorescence signal is detected, after the target is imprinted. (2) The substance to be detected with the FIP is labeled, and the fluorescence signal of the remaining labeled substance in the solution is determined by competing with the binding site of the fluorescent label to bind the MIPs. The indirect MIFs [53,54] have been widely reported in the literature.

### 3.2. Sensor Classification

**Figure 3:**
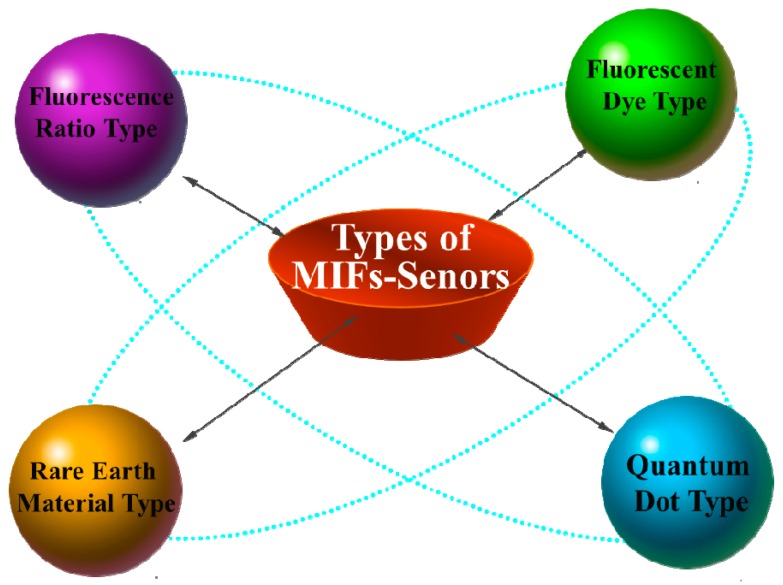
Four types of MIFs based on different fluorescence signals sources.

The MIFs is an organic combination of molecularly imprinted technology and fluorescent nanomaterials. Therefore, these sensors can demonstrate the dual advantages of highly sensitive detection of fluorescence probes and specific recognition adsorption of MIP using the variations in the fluorescence signals. This compensates for the drawback of the MIP, which can only recognize and not transmit the signal out of the defect. According to the different sources of fluorescence signals, indirect MIFs can be divided into four types (presented in Figure 3): organic fluorescent dye type, quantum dot type, rare earth material type, and ratio fluorescence type.

#### 3.2.1. Organic Fluorescent Dye Type

Common organic dyes include fluorescein isothiocyanate (FITC) [55], 5-(4, 6-dichlorotriazine) aminofluorescein (5-DTAF) [56], rhodamine [57], and so on. Liu et al. synthesized magnetic MIPs with good adsorption selectivity and stress resistance by precipitation polymerization using magnetic chitosan nanoparticles as functionalized carriers and atrazine as template molecules. Using 5-DTAF as a fluorescence probe for atrazine structural analogues, a fluorescence competitive atrazine biosensor based on the magnetic molecularly imprinted chitosan surface was constructed [58]. In addition, Liu et al. obtained FITC-Mel fluorescent labeling molecule by modifying FITC to melamine molecules. FITC-Mel and Mel compete for recognition sites on the surface of the magnetic core-shell MIPs. Fluorescence sensors for melamine in milk and infant milk powder were established by detecting the fluorescence intensity of FITC-Mel in the respective solutions. The linear range is 0.1–20.0 mg/Kg and the detection limit is 0.05 mg/Kg [59].

#### 3.2.2. Quantum Dot Type

Quantum dots (QDs) are semiconductor nanocrystals with a three-dimensional structure of nanometer scale (1–10 nm) and a size radius of the exciton bohr radius. The quantum dots are used as carriers in the fluorescence signal unit. The quantum dot-imprinted polymers (QDs-MIPs) synthesized by imprinting polymerization on their surfaces possess both the advantages of surface molecular imprinting and the high sensitivity and selectivity of QDs. It is important to integrate the recognition unit and signal output unit efficiently, as it demonstrates broad application prospects [60,61].

**Figure 4:**
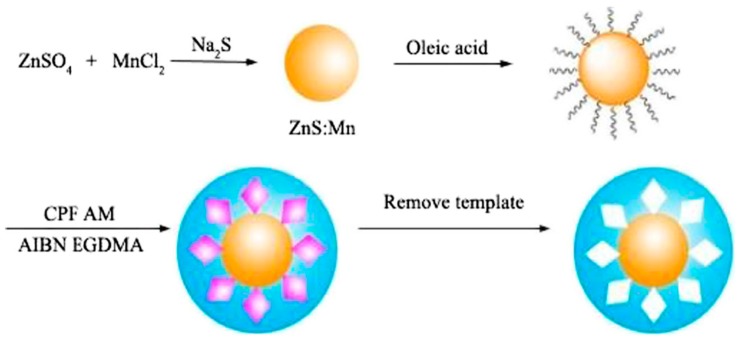
The preparation process of MIPs-coated quantum dots (QDs). Reprinted with permission from [63], copyright (2015) Springer Nature.

Zhang et al. modified the ionic liquids to CdSe/ZnS QDs based on electrostatic interaction, interacted with carbaryl and methacrylic acid, deposited a MIP on the surface of CdSe/ZnS QDs, and finally obtained molecularly imprinted quantum dot fluorescent probes. The linear range of carbaryl was 0.0398–49.7 μM, and the detection limit was 14.7 nM [62]. Ren et al. used the ZnS QDs doped with Mn (Ⅱ) for the synthesis of MIPs with acrylamide as the functional monomer, ethylene glycol dimethacrylate as the crosslinking agent, and azobisisobutyronitrile as the initiator and the preparation process of MIP-coated QDs is shown in Figure 4. The fluorescence sensing system was applied for determining the organophosphorus pesticide chlorpyrifos residues with a detection limit of 17 nM [63]. Zhao et al. synthesized MIPs composite nanomaterials based on QDs by ultrasonic-assisted method for the determination of organophosphorus pesticide diazinon residues. Due to the non-covalent interactions between the polymer matrix and template molecules, this material exhibits good specificity in aqueous media. The detection principle included the mechanism of energy transfer between the quantum dots and target molecule diazinon, which led to fluorescence quenching. The fluorescence quenching degree of quantum dots was proportional to the concentration of diazinon. The linear range was 50–600 ng/mL, and the detection limit was 50 ng/mL [64].

In recent years, MIFs based on carbon QDs [65], graphene QDs [66], and C_3_N_4_ QDs [67] have been developed. These fluorescence sensors overcome the problem of toxic elements overflowing from the semiconductor quantum dots. They are also eco-friendly and generate a strong fluorescence signal [68]. Liu et al. prepared silylated carbon QDs by performing the hydrothermal treatment of aminosilylation reagent and citric acid. Then, carbon QDs-MIPs were synthesized by sol-gel method and used for fluorescence detection of bisphenol A [69]. Hassanzadeh et al. introduced a molecularly imprinted layer on the surface of C_3_N_4_ QDs by sol-gel blotting. When amikacin specifically bound to the recognition sites of MIPs, the fluorescence of C_3_N_4_ QDs was quenched. In the concentration range of 4.4–585.1 nM, the MIFs demonstrated good linearity [67].

#### 3.2.3. Rare Earth Material Type

The rich orbital energy levels of rare earth ions and the transition characteristics of 4f electrons enable rare earth nanoparticles, rare earth complexes, rare earth doped upconversion nanoparticles, and other rare earth materials to become potential novel luminescent (fluorescent) materials [70]. Rare earth materials have attracted considerable attention in the optical field because of their high anti-Stokes shift, narrow-band emission, long luminescence lifetime, high optical/chemical stability, and low biological toxicity [71]. MIPs synthesized by rare earth materials demonstrate the advantages of rare earth luminescent materials as well as MIPs. The MIFs with anti-interference and anti-photobleaching properties possess good application prospects [72,73], because they can produce changes in the fluorescence characteristics of rare earth materials, when the target specifically binds to the MIPs.

**Figure 5:**
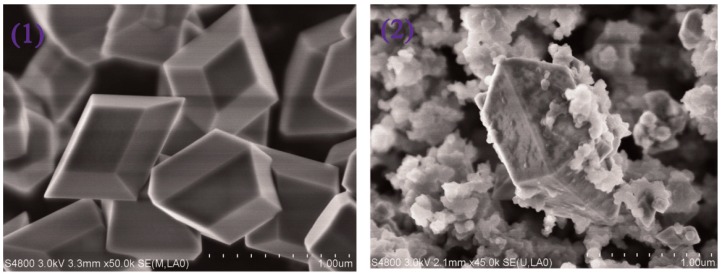
Scanning electron microscope (SEM) structure images of (**1**) upconverting particles (UCPs), and (**2**) MIPs@UCPs. Reprinted with permission from [74], copyright (2015) Elsevier.

Tang et al. synthesized YF_3_: Yb^3+^ Er^3+^ upconverting particles (UCPs) using the hydrothermal method. The structure of MIPs@UCPs is presented in Figure 5, which demonstrates that the imprinting sites were successfully coated onto the surface of UCPs. The clenbuterol derivatives were used as template molecules, methacrylic acid was used as the functional monomer, and ethylene glycol dimethacrylate was used as the crosslinking agent. Molecular imprinting upconversion fluorescent probes with selective recognition ability for clenbuterol were synthesized. The clenbuterol molecules entered the imprinting site and quenched the upconversion fluorescence intensity, when the probe was placed in the solution. The linear range of clenbuterol was 5.0–100.0 μg/L, and the detection limit was 0.12 μg/L. Finally, the water and pork samples were analyzed using the constructed probe, and the results showed good reproducibility, which could effectively avoid false positive results [74]. Zheng et al. prepared a core-shell fluorescence sensor based on monodisperse particles coated with silicon by hydrolysis of tetraethyl orthosilicate. The core-shell fluorescence sensor was prepared by the copolymerization of methacrylic acid and ethylene glycol dimethacrylate using monodisperse particles of approximately 200 nm as the carrier and copper ions as the template. The fluorescence detection of Cu^2+^ was realized with a linear range of 10–100 μmol/L [75]. Tang et al. prepared upconversion fluorescent molecularly imprinted polymers (UFIPs) on NaYF_4_: Yb^3^+ Er^3+^ nanoparticles modified using layer-by-layer self-assembly strategy. The UFIPs were prepared by radical initiation polymerization. The imprinted polymers can be used for the fluorescence analysis of five quinolones residues in fish meat with a detection limit of 1.03 nM to 0. 30 μM [76].

#### 3.2.4. Ratio Fluorescence Type

The ratio fluorescence method is an analytical method to determine the target substance by measuring the fluorescence intensity at two different wavelengths and considering the ratio as the signal parameter [77]. This method can use the fluorescence intensity ratio variations to improve the dynamic response range, improve the accuracy and sensitivity of determination, and ultimately achieve accurate quantitative determination of the sample [78]. This method was introduced in the synthesis process of MIFs, and the constructed molecularly imprinted ratio fluorescence sensor can enhance the detection sensitivity and anti-interference ability [79]. The ratio molecularly imprinted materials comprise two fluorescent materials with different emission peaks. One of the fluorescent materials interacts directly with the target and changes its fluorescence intensity, while the other material maintains the same fluorescence intensity as a reference. The fluorescence emission signals of the two materials are measured simultaneously under the excitation of a single wavelength, and the fluorescence intensity ratio is used to quantitatively detect the target. Ratio MIFs can self-calibrate the detection parameters, thus reducing or even eliminating the influence of interference factors and rendering the analysis results more accurate [80].

**Figure 6:**
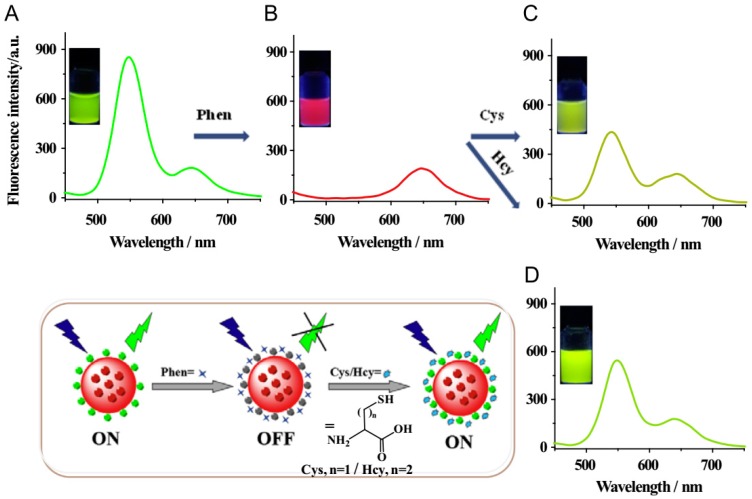
Fluorescence spectra of the dual-emission rQDs@SiO_2_@gQDs Hybrid spheres and the working principle for the visual fluorescence detection of cysteine and homocysteine: (**A**), hybrid spheres–Phen (30 μM) (**B**), hybrid spheres–Phen (30 μM)–Cys (1000 μM) (**C**), hybrid spheres–Phen (30 μM)–Hcy (800 μM) (**D**). Reprinted with permission from [82], copyright (2015) Elsevier.

Yao et al. encapsulated red fluorescent CdTe QDs into silicon spheres and covalently attached green fluorescent CdTe QDs to the surface of silicon spheres to construct ratio fluorescence sensors. The encapsulated red fluorescent QDs were not sensitive to the target Cu^2+^, while the green fluorescent CdTe QDs in the presence of Cu^2+^ quenched the fluorescence intensity. The detection limit of Cu^2+^ was as low as 1.1 nM. The method was successfully applied for the determination of Cu^2+^ in lake water, mineral water, and grass samples [81]. Wang et al. considered the red fluorescent CdTe QDs composite material coated with silicon as a reference signal. The green fluorescent CdTe QDs that covalently bonded on the surface of silica spheres were used as the response signal. An on-off-on ratio fluorescence sensor was successfully constructed using the fluorescence intensity change (fluorescence quenching-fluorescence recovery) of red CdTe QDs and green CdTe QDs, which could be used to detect cysteine and homocysteine in situ [82]. Figure 6 depicts fluorescence spectra of the dual-emission rQDs@SiO_2_@gQDs Hybrid spheres and the working principle for the visual fluorescence detection of cysteine and homocysteine.

## 4. Application of MIFs in Food Quality and Safety Detection

Currently, the determination of food quality and safety hazard factors mainly involves chromatography techniques, such as high-performance liquid chromatography, gas chromatography, and high-performance liquid chromatography-mass spectrometry. These methods demonstrate the advantages of high recovery, good reproducibility, and low detection limit; however, they frequently require a tedious sample pretreatment process. Moreover, there are other drawbacks, such as expensive equipment and reagents, long detection time, unsuitable for detection of a large number of samples, and lack of portability. Fluorescence sensing analysis demonstrates the characteristics of high sensitivity, low detection limit, fast reaction speed, good selectivity, low cost, and usage of relatively simple instruments and equipment. In recent years, with the gradual progress and maturity of surface molecular imprinting, nanomolecular imprinting technology, and high-performance fluorescent nanomaterials preparation technology, the selective recognition and fluorescence detection performance of MIFs with respect to hazard factors in complex food matrices have been significantly enhanced [83]. The MIFs have been widely used in food quality and safety analysis. Most of the studies focus on the fluorescence detection of agricultural and veterinary drug residues [9,84], drug residues [85], prohibited additives [86], heavy metals, environmental organic pollutants, and other hazard factors.

### 4.1. Pesticides

The long-term and large-scale use of pesticides leads to environmental pollution and increased pesticide residues in agricultural products, thereby destroying the ecological balance, increasing the risk of food safety, and endangering human health [87]. The fluorescent MIPs prepared by molecular imprinting technique is a three-dimensional crosslinked polymer with specific recognition sites, which can realize selective recognition, adsorption, and fluorescence detection of pesticide molecules (template molecules).

**Figure 7:**
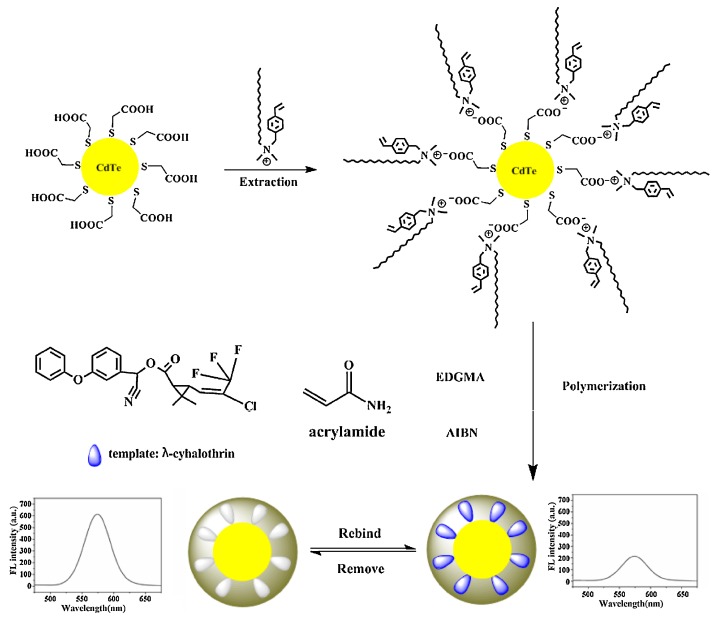
The preparation principle of fluorescent molecularly imprinted polymers (FIPs) for recognizing cyhalothrin. Reprinted with permission from [89], copyright (2016) Elsevier.

Li et al. [88] prepared MIPs with specific fluorescence response to cyhalothrin by silylation of FeSe QDs. These MIPs demonstrate good linearity in the concentration range of 0.010–0.20 mg/L and a detection limit of 1.3 μg/L in fish meat products. Using surfactant-modified CdTe QDs as fluorescence signal source as well as carrier and acrylamide as the functional monomer, Wei et al. [89] prepared FIPs as shown in Figure 7, which can specifically recognize cyhalothrin using free radical polymerization. In the concentration range of 0.1–16 μM, when MIPs were combined with cyhalothrin, the fluorescence of the biosensor was turned off, and the rapid fluorescence analysis of cyhalothrin residues in Yangtze River water was realized. Wang et al. [90] used SiO_2_-coated red QDs as the support carrier and reference signal source. The green fluorescent dye 4-amino-7-nitro-N-octylbenzo (1,2,5) oxadiazole was used as the recognition signal, 3-aminopropyltriethoxysilane (APTES) was used as the functional monomer, and 2, 4-dichlorophenoxyacetic acid (2, 4-D) was used as the template molecule. The imprinted layer was prepared using the sol-gel method. With the increase of 2, 4-D concentration, the ratio fluorescence intensity changed, and the fluorescence color changed from orange-red to green, thus realizing the fluorescence analysis of 2, 4-D.

Wagner et al. successfully constructed a fluorescence sensing platform for nanomolar concentration of 2, 4-D pesticide residues with the deposition of organic dye-encapsulated MIPs on the surface of silicon spheres using the surface imprinting method and incorporation of the prepared fluorescent MIPs into microfluidic analysis techniques [8]. According to Liu et al. [91], FIPs encapsulated nitrogen-doped graphene QDs were prepared by alkaline self-polymerization of dopamine using dopamine as the functional monomer and crosslinking agent. The FIPs were deposited on the surface of the test strip by the adsorption of the filter strip, and a fluorescent strip was constructed for specific detection of thiamethoxam. Amjadi et al. prepared a ratio MIFs by imprinting carbon quantum dot silica spheres and CdTe/CdS QDs in the same polymer using the sol-gel method. The fluorescence intensity of CdTe/CdS QDs decreased and the fluorescence intensity of CDs QDs remained unchanged, when the sensor was combined with diniconazole. The linear range of the determination was 20–160 μg/L, and the detection limit was 6.4 μg/L for diniconazole residues in environmental water and soil samples [80].

### 4.2. Veterinary Drugs/Drug Residues

In recent years, due to the illegal use of prohibited additives, improper use of veterinary drugs as well as overuse of medical drugs in production, use of livestock, poultry, and aquatic products, and residues of veterinary drugs, medical drugs, and prohibited additives have become some of the important factors affecting food safety. For example, tetracycline is widely used in livestock and poultry production as a broad-spectrum bacteriostatic agent. Due to the high dosage, long time, and abuse of the drug, it produces serious residues in animal muscle, milk, liver, and other foods, thereby endangering food safety [92]. Based on high selectivity and good stability, FIPs can efficiently recognize and detect veterinary drug molecules in complex sample environments (meat products, animal blood, urine, and feces) to cope with a variety of adverse factors. Therefore, the veterinary drug residue analysis method based on MIFs demonstrate higher sensitivity and selectivity.

Using allyl fluorescein as the fluorescent functional monomer, Wang et al. [93] prepared FIPs with specific response to tetracycline using the surface imprinting method and applied them to the fluorescence analysis of tetracycline in human serum and pig urine. Using malachite green as the template molecule, (3-Aminopropyl)triethoxysilane (APTES)as the functional monomer, and tetraethoxysilane (TEOS) as the crosslinking agent, Wu et al. [94] prepared a MIFs for specific recognition of malachite green by embedding CdTe QDs. The fluorescence intensity of CdTe QDs at 370 nm decreased rapidly with an increase in the malachite green concentration. The detection limit of CdTe QDs in fish meat was 12 μg/L. Ming et al. prepared the magnetic surface MIPs of estradiol using surface imprinting and free radical polymerization. The estradiol and fluorescent labels competed for the binding sites of adsorption MIPs. After the magnetic separation, estradiol residues could be quantitatively detected by measuring the fluorescence intensity of fluorescent labels in the solution. This technique does not require fluorescent quantum dots or fluorescent monomers to be embedded in the MIPs, and the detection process is simple and rapid [95].

**Figure 8:**
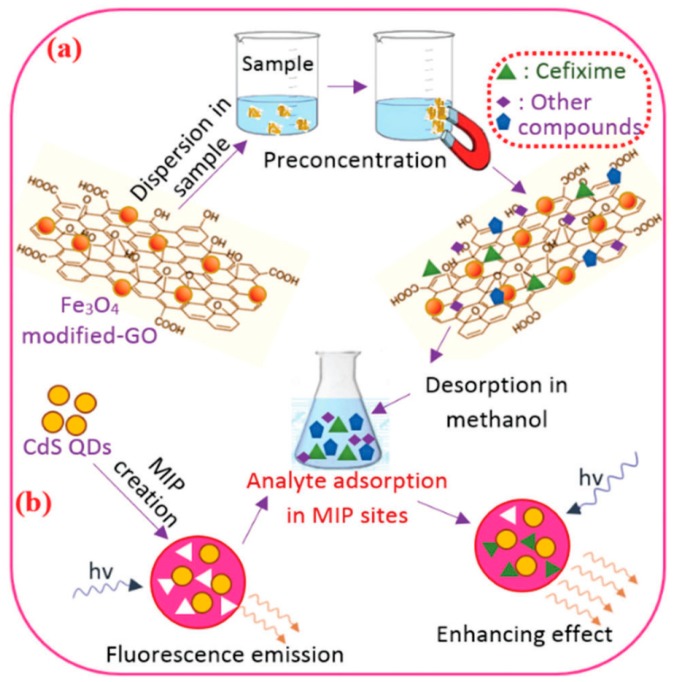
The preparation of FIPs based on CdS quantum dots for the determination of cefixime residues in urine: (**a**) the preconcentration of cefixime, (**b**) fluorometric determination using FIPs. Reprinted with permission from [96], copyright (2017) Royal Society of Chemistry.

Mehrzad-Samarin et al. reported a method for the preparation of graphene QDs in MIPs using the hydrothermal method and sol-gel method. When the fluorescent molecule imprinted specifically bound to the template molecule metronidazole, the fluorescence of the graphene quantum dot was “Turn-Off.” An MIFs for the determination of metronidazole in plasma was successfully constructed with a linear range of 0.2–15.0 μM and a detection limit of 0.15 μM [53]. As shown in Figure 8, the FIPs based on CdS quantum dots were successfully prepared by Eskandari et al. and applied for the determination of cefixime residues in urine with a linear range of 0.001–0.7 μg/mL and a detection limit of 0.54 ng/mL [96]. The present method was successfully used to determine the concentration of cefixime residues in pharmaceutical and urine samples. The results were shown to possess good specificity for cefixime in the presence of other interferences.

### 4.3. Heavy Metals

Due to the rapid development of modern industrial economy, the discharge of heavy metal sewage is becoming increasingly serious and poses a significant amount of threat to the environment and biological health. Heavy metals are highly toxic, non-degradable, bioaccumulative, and easy to enrich and transfer in the food chain. They pollute the agricultural products and food causing serious harm [97]. In view of the heavy metal pollution, the development of a simple, rapid, and accurate MIF is of great significance to protect the environment of agricultural products, food quality, and safety.

Wang et al. [98] coated red CdTe QDs into aminosilicone spheres, coupled carboxylated CdSe QDs onto the surface of aminosilicone spheres by 1-ethyl-3-(3-dimethylaminopropyl)carbodiimide (EDC)/N-hydroxysuccinimide (NHS) method, and then prepared double-excited fluorescence ion imprinting with a specific binding ability to Cd^2+^ using ethylenediaminetetraacetic acid (EDTA) etching. The imprinted material emits red fluorescence before binding Cd^2+^ and green fluorescence after binding Cd^2+^. The ion-imprinted fluorescence sensor demonstrated good linearity in the concentration range of 0.1–9 μM with a detection limit as low as 25 nM. Luo et al. [99] directly introduced CdTe QDs on the surface of sulfhydryl-modified magnetic silica spheres and prepared fluorescent ion imprinting using EDTA etching. The imprinting could be used not only for the fluorescence quantitative analysis of Cd^2+^ but also for magnetic adsorption and removal of Cd^2+^. The maximum adsorption capacity of Cd^2+^ was as high as 17.57 mg/g. Using 8-hydroxyquinoline as fluorescent functional monomer, Tan et al. [100] prepared Zn^2+^ and Cd^2+^ ion imprinting by sol-gel blotting. The imprinted fluorescence sensor demonstrated good selectivity and high adsorption capacity, because the fluorescence intensity decreased, when the imprinted material bound the target ions. Ion-imprinted polymers with specific fluorescence response to Al^3+^ were prepared by radical polymerization using 8-hydroxyquinoline-5-sulfonic acid as the fluorescent functional monomer [101]. The fluorescent ion imprinting demonstrated good anti-interference (Cu^2+^, Zn^2+^) and still exhibited high adsorption capacity for Al^3+^ after nine times of reuse.

The fluorescent multifunctional monomer was synthesized by Sun et al. to specifically bind Ag^+^ and generate free radical polymerization with a crosslinking agent. The fluorescence at 490 nm was “Turn-Off,” and the fluorescence intensity was negatively correlated with the concentration of Ag^+^ [102].

### 4.4. Environmental Organic Pollutants

Polycyclic aromatic hydrocarbons, phenols, organic dyes, and other environmental organic pollutants are widely distributed and contain toxic, teratogenic, and carcinogenic elements, which are considered important threats to human health. In previous research, the high-performance liquid chromatography (HPLC) and mass spectrometry (MS) were often used to determine the content of urinary albumin. Although the sensitivity and specificity of MS were high, the equipment was expensive, and the detection cost was high. MIFs have been widely employed for rapid detection of organic pollutants in the environment.

Li et al. [97] prepared a FIPs by encapsulating YVO_4_:Eu^3+^ rare earth nanoparticles and carbon quantum dots. When p-nitrophenol was adsorbed on MIPs, the fluorescence of carbon quantum dots was quenched, while the fluorescence of YVO_4_:Eu^3+^ rare earth nanoparticles remained unchanged. Based on the above principle, a ratio fluorescence sensor was constructed and applied for the rapid determination of p-nitrophenol in environmental water and urine with a detection limit of 0.15 μM. Zhou et al. [103] hydrothermally treated APTES-grapheme oxide to obtain silylated graphene quantum dots, which were introduced into the FIPs of p-nitrophenol by sol-gel method. In the concentration range of 0.02–3.0 μg/mL, the MIPs fluorescence sensor could be effectively quenched by p-nitrophenol with a detection limit of 9.0 ng/mL. Wu et al. [104] reported the preparation of FIPs using AuCNs for the first time and successfully applied these FIPs in the fluorescence analysis of bisphenol A in seawater. APTES was used as the functional monomer, while TEOS was used as the crosslinking agent to deposit the molecularly imprinted layer on the surface of SiO_2_ @ AuCNs (obtained by modifying carboxyl-terminated AuNCs onto the surface of amino-SiO_2_ nanoparticles, which are defined as SiO_2_ @ AuCNs). The fluorescence intensity of MIPs decreased with an increase in the BPA concentration to 396 nm while the detection limit was 0.1 μM. Qi et al. [46] combined the ratio fluorescence molecularly imprinted polymerization with microfluidic technique to construct a paper chip for fluorescence detection of phenol-based environmental pollutants. They modified carboxyl CdTe QDs on cellophane using the EDC/NHS method and successfully prepared fluorescent molecularly imprinted paper chips, which could bind 4-nitrophenol and 2, 4, 6-trinitrophenol simultaneously using the double-template imprinting and sol-gel imprinting techniques. The detection limits in the environmental water samples were 0.097 and 0.071 mg/L. The analytical performances of MIFs in determination of pollutants in food safety are summarized in Table 1.

However, the real sample analysis based on MIFs often suffered from the complicated matrix interference and which will result in false positive and false negative results. It is necessary to develop a novel strategy to construct anti-interference FIPs and MIFs for monitoring trace pollutants in food and environment samples. In addition, combining a rapid pretreatment technique with MIFs is another effective solution to avoiding matrix interference.

## 5. Conclusions and Prospects

MIFs combine the high selectivity of MIT with the high sensitive response of fluorescent materials and then convert the molecular recognition into readable fluorescence signal. This aspect compensates for the drawback of the MIPs that can only recognize and not transmit the signal and integrates the recognition unit and signal output unit efficiently. The MIFs further improve the performance of molecular imprinting and broaden its range of applications, thus promoting high-efficiency enrichment and high-sensitivity detection of trace substances in complex matrices. Compared to traditional analytical techniques, MIFs exhibit the characteristics of high sensitivity and high selectivity and therefore demonstrate significant potential and good application prospects with respect to the rapid detection of food and environment safety.

According to the different sources of fluorescence signals, there are four types of MIFs. Organic dye type of MIFs is obtained by embedding common organic dyes into MIPs. Although the preparation is simple, and the detection process is rapid, organic dyes are easy to be bleached by interference substances from the complicated matrix. QDs are often used as fluorescence signals to synthesize FIPs and MIFs because of their unique properties, involving high luminous efficiency, stability, and narrow emission spectra. However, QDs types of MIFs are difficult and time-consuming to prepare. More importantly, most QDs are poisonous to human health and threaten environmental ecology. The preparations of rare earth material types and ratio fluorescence types of MIFs are involving many synthetic steps, which will cause the instability of the FIPs structure and generate false detection results. In consequence, various novel MIFs still encounter many challenges during the development process.

The MIT provides a good approach for the specific detection of targets; however, the related research is still imminent, and the technology is not entirely developed [105,106]. Therefore, mass production with MIT has not been achieved so far. Firstly, the FIPs prepared by the existing synthesis methods still suffer some drawbacks, such as irregular shape, heterogeneous particle size, non-uniform interaction sites, and long polymerization time. Therefore, it is necessary to develop new synthesis methods with respect to MIPs. Secondly, the research on the molecular imprinting process, molecular recognition mechanism, mass transfer mechanism, and the characterization of polymer structure is still highly limited and requires further developments. Further research is required to increase the selectivity, mass transfer rate, and adsorption capacity of molecularly imprinted materials as well as prepare functional monomers that can specifically bind to the template molecules instead of universal monomers. In addition, the presence of two or more residues in the food matrix is very common, frequently requiring multiple detections of varied contaminant residues in the same sample. Therefore, it is an important direction for the future development of MIFs that can simultaneously detect multiple food safety factors by adjusting the probe size, element composition, and synthesis methods and develop a composite MIFs. To sum up, designing and developing more functional monomer-template interaction systems, conducting an in-depth study of controllable polymerization methods, preparation of MIFs with improved selectivity, thinner polymeric layer and higher sensitivity, and the combination of smart phones, tablet computers, and cloud databases to build a new intelligent fluorescence rapid detection platform are of great significance in promoting the development of rapid detection technology for food and environment safety.

## Figures and Tables

**Table 1:** The analytical performances of MIFs in determination of pollutants in food and environment samples.

Type of Food Contaminants	Fluorescent Sources of MIFs	Analytes	Samples	LOD	References
Pesticides	CdSe/ZnS QDs	Carbaryl	Chinese cabbage	14.7 μM	[62]
	Mn-doped ZnS QDs	Chlorpyrifos	River water samples	17 nM	[63]
	CdTe/CdS QDs- CDs	Diniconazole	Water and soil samples	19.6 nM	[80]
	FeSe QDs	Cyhalothrin	Fish meat	1.4 nM	[88]
	N-GQDs	Thiamethoxam	Water samples	0.1 μM	[91]
Heavy metal	Eu(TTA)_3_phen	Cu^2+^	Water and biological samples	-	[75]
	Ratio CdTe QDs	Cu^2+^	Lake water, mineral water, and grass samples	1.1 nM	[81]
	CdSe QDs	Cd^2+^	Water samples	25 nM	[98]
	Magnetic CdTe QDs	Cd^2+^	Water samples	-	[99]
	Functional monomer	Ag^+^	Water samples	10 μM	[102]
Drug residues	GQDs	Metronidazole	Plasma matrixes	0.15 μM	[53]
	C_3_N_4_ QDs	Amikacin	Biological samples	1.8 nM	[67]
	YF_3_: Yb^3+^ Er^3+^	Clenbuterol	Water and pork samples	0.42 nM	[74]
	Allyl fluorescein-SiO_2_	Tetracycline	Human serum and pig urine	4.26 nM	[93]
	CdTe QDs	Malachite green	Fish meat	12.9 nM	[95]
Environmental pollutants	CdTe QDs	4-nitrophenol and 2, 4, 6-trinitrophenol	Environmental water samples	0.7 μM; 0.31 μM	[46]
	CDs	Bisphenol A	River water samples	30 nM	[69]
	YVO_4_: Eu^3+^	p-Nitrophenol	Water samples	0.15 μM	[97]
	GQDs	p-Nitrophenol	Water samples	39.4 nM	[103]
	AuCNs	Bisphenol A	Sea water	0.1 μM	[104]
